# Comparing the Therapeutic Effects of Carvedilol and Metoprolol on Prevention of Atrial Fibrillation after Coronary Artery Bypass Surgery, a Double-Blind Study

**Published:** 2014-09-01

**Authors:** Rozita Jalalian, Rahman Ghafari, Peyman Ghazanfari

**Affiliations:** 1 Department of Cardiology, Faculty of Medicine, Mazandaran University of Medical Sciences, Sari, IR Iran

**Keywords:** Atrial Fibrillation, Carvedilol, Metoprolol

## Abstract

**Background:**

Atrial Fibrillation (AF) is a common complication after open heart surgery and is frequently associated with increased hospital stay, complications, and mortality rates. The effect of β-blockers on prevention of supraventricular arrhythmias has been confirmed in several prospective randomized studies.

**Objectives:**

This clinical trial aimed to compare the preventive effects of carvedilol and metoprolol on occurrence of AF after CABG surgery.

**Patients and Methods:**

This prospective, double-blind, randomized clinical trial was conducted on 150 patients (55 females, 95 males; mean age: 59 ± 10 years) who underwent CABG surgery. The patients with no contraindication for β-blocker use were randomly divided into two groups of carvedilol and metoprolol Tartarate (n = 75). Treatment with β-blocker was started on the first postoperative day (metoprolol, 25 mg BD; carvedilol, 6.25 mg, BD) and the dosage was regulated according to the patients’ hemodynamic response. All the patients were monitored 5 days after the surgery and incidence of AF and other complications was recorded in both groups.

**Results:**

AF was detected in 18 patients in the carvedilol group and 21 patients in the metoprolol group (P = 0.577). The results of Fisher Exact test showed no significant relationship between the type of the drug and the occurrence of AF (P < 0.05). Nevertheless, the prevalence of AF was higher in the renal failure group. AF was mostly recorded on the second and third days after the surgery. The results showed an association between old age and higher occurrence of AF. AF was recorded in 11 patients (14%) in the metoprolol group and 9 ones (12%) in the carvedilol group, with Left Ventricle Ejection Fraction (LVEF) being between 35% and 45% (P = 0.587). However, no significant difference was found between the two groups in this regard.

**Conclusions:**

In the patients with sufficient ejection fraction, no difference was observed in using carvedilol or metoprolol in prevention of post-CABG AF. Yet, given the anti-oxidant and anti- inflammatory effects of carvedilol, it might be more beneficial in comparison to metoprolol, particularly in the patients with lower ejection fractions or heart failure.

## 1. Background

Coronary Artery Bypass Grafting (CABG) surgery is commonly associated with complications, such as supra-ventricular and ventricular arrhythmias (1). Atrial Fibrillation (AF) is the most common arrhythmia after CABG (2) which is associated with hemodynamic disturbances, systemic emboli, higher incidence of Myocardial Infarction (MI), and complications due to anti-coagulant administration, including pericardial effusion and tamponade. Moreover, it increases the length of hospital stay, costs, and mortality rate, and complicates the post-operative period (3, 4). It has an incidence rate of 20 - 40% and usually occurs between the third and fifth days after the surgery (4). Several factors are known to affect the incidence of AF after CABG, including old age, left ventricular failure, history of previous AF, Chronic Obstructive Pulmonary Diseases (COPD), previous MI, size of the left ventricle, history of β-blocker use, cross clamp time during the surgery, and CPB time (2). The efficacy of β-blockers in prevention of AF after CABG has been confirmed in several clinical trials and meta-analyses (5, 6). Moreover, using β-blockers after MI has been shown to be associated with decreased rate of sudden death, recurrent MI, ventricular fibrillation, and cardiogenic shock (7). Metoprolol is a selective blocker of β1 receptor which has been proved to be effective in reducing the incidence of AF after CABG (8, 9). Carvedilol is also a non-selective blocker of β and α1 adrenergic receptors (1). Recent data have suggested different pharmacological characteristics of carvedilol in comparison to other β- blockers, such as anti-inflammatory and anti-oxidant properties (1). In the patients with MI and systolic dysfunction of the left ventricle, carvedilol prevents the remodeling of the left ventricle and thus decreases mortality and morbidity rates. In addition, it increases the ejection force of the left ventricle and reduces end-diastolic volume in the patients with heart failure, particularly severe cases. It is also well known as an effective medication in improvement of mortality, length of hospital stay, and clinical manifestations of heart failure (8). In addition to being an alpha and beta receptor blocker, carvedilol can also be classified as a class II anti-arrhythmic agent (10).

## 2. Objectives

Due to the lack of any definite preventive strategies for AF following CABG surgery, this clinical trial aims to compare the preventive effects of carvedilol and metoprolol on CABG patients.

## 3. Patients and Methods

In this prospective, double-blind, randomized clinical trial, post- CABG patients admitted in ICU were randomly assigned to carvedilol or metoprolol Tartarate groups using codes. The data were first recorded in questionnaires and were then correlated with the patients’ codes. The study population consisted of the patients indicated for CABG surgery who had referred to Fatemeh-Zahra Hospital and did not have any contraindications for beta blocker use. These contraindications included chronic obstructive respiratory failure, advanced AV block, previous AF, bradycardia (HR < 60), hypotension (SBP < 90), ejection fraction < 35%, and having undergone repair or replacement of the heart valve along with their CABG surgery. Overall, 150 patients (95 males and 55 females between 21 and 81 years old) were enrolled into the study (75 patients in each group).

### 3.1. Operation Data

In this study, CABG surgery wass performed in two forms, namely cardiopulmonary bypass (CPB) and off-pomp CABG. In CPB method, medial sternotomy was performed. After sufficient heparinazation and taking a graft, pericardium was cut open and CPB was established by cannolization of the ascending aorta and the right atrium. All the patients were put under moderate hypothermia (28 - 30C). Then, distal and proximal anastomoses were repaired during aortic clamp and after removing the aortic cross clamp, respectively. The patients were warmed afterwards. Cardiac monitoring was performed in all the patients during post-operative admission to the cardiac care unit.

### 3.2. Data Gathering

All the patients were informed about the study protocol and written informed consents were obtained from the patients. Then, post-CABG patients were randomly assigned to two groups of treatment with metoprolol and carvedilol according to the predetermined codes (neither the researchers nor the patients were informed). The initiative dose was 25 mg/BD in the metoprolol group and 6.25 mg/day in the carvedilol group. The dose was increased according to the patients’ hemodynamic responses. Cardiac monitoring was performed in all the patients during ICU and 5 days post-ICU and the patients were assessed by the research team if any AF or VF arrhythmias occurred. AF criteria included absence of P wave or irregular QRS complex continued for at least 30 seconds. The patients with AF were treated by anti-coagulant agents and amiodarone or cardioversion. Eventually, all the data related to before, during, and after the surgery were recorded in the questionnaires. Pre-operative data included age, gender, history of hypertension, diabetes, renal insufficiency, chronic obstructive lung diseases, previous beta blocker use, previous MI, ejection fraction, and left atrium diameter. Besides, peri-operative data included type of the CABG surgery, CPB duration, cross clamping time, and type of the grafting artery (distal anastomosis). Finally, post- operative data included occurrence of AF, day of AF occurrence, maximum ventricular rate, and occurrence of ventricular fibrillation. After all, the data were entered into the SPSS statistical software (v. 17) and analyzed using Fisher exact test and independent samples T-Test.

## 4. Results

Among the 150 studied patients, 95 (63%) were male and 55 (37%) were female with the mean age of 59 ± 10 years. The patients were randomly assigned to two groups of 75 being treated with either carvedilol or metoprolol (60 ± 10 carvedilol, 57 ± 10 metoprolol).

The results revealed no significant difference between the two groups regarding demographic parameters, ejection fraction, and left atrium diameter (Table 1). Pre-CABG data were also similar, except for the incidence of renal insufficiency which was detected in 5.3% of the patients in the metroprol group, but was not observed in the carvedilol group (P < 0.05). Also, no significant difference was found between the two groups concerning the peri-operative data, such as type of the surgery, CPB time, cross clamping time, and type of the anastomosed artery (Table 2).

**Table 1.** Pre-Operative Data in Carvedilol and Metoprolol Groups

Pre-Operative Data	Carvedilol (n = 75)	Metoprolol (n = 75)	P value
**Age (year)**	60 ± 10	57 ± 10	0.151
**Male gender (number)**	47 (63%)	48 (64%)	0.865
**Hypertension (number)**	67 (89%)	65 (87%)	0.615
**Diabetes (number)**	31 (41%)	38 (50%)	0.256
**Previous MI (number)**	30 (40%)	26 (35%)	0.501
**Renal failure (number)**	0 (0%)	4 (5.3%)	0.043
**Previous β blocker use (number)**	71 (95%)	70 (93%)	0.731
**Left ventricular ejection fraction (percent)**	45.5 ± 7%	47 ± 8%	0.179
**Left atrial diameter (millimeter)**	39 ± 4.3	39 ± 4.4	1.000

**Table 2.** Peri-Operative Data in Carvedilol and Metoprolol Groups

Peri-Operation Data	Carvedilol (n = 75)	Metoprolol (n = 75)	P value
**On-pump surgery (n)**	70 (93%)	72 (96%)	0.467
**CPB time (min)**	83 ± 52	82 ± 23	0.927
**Cross clamping time (min)**	44 ± 23	46 ± 18	0.555
**Number of anastomosis**	1.68 ± 0.49	1.78 ± 0.4	0.155
**ITA graft**	63	55	-
**RA graft**	8	3	-
**SV graft**	63	65	-

**Figure 1.**
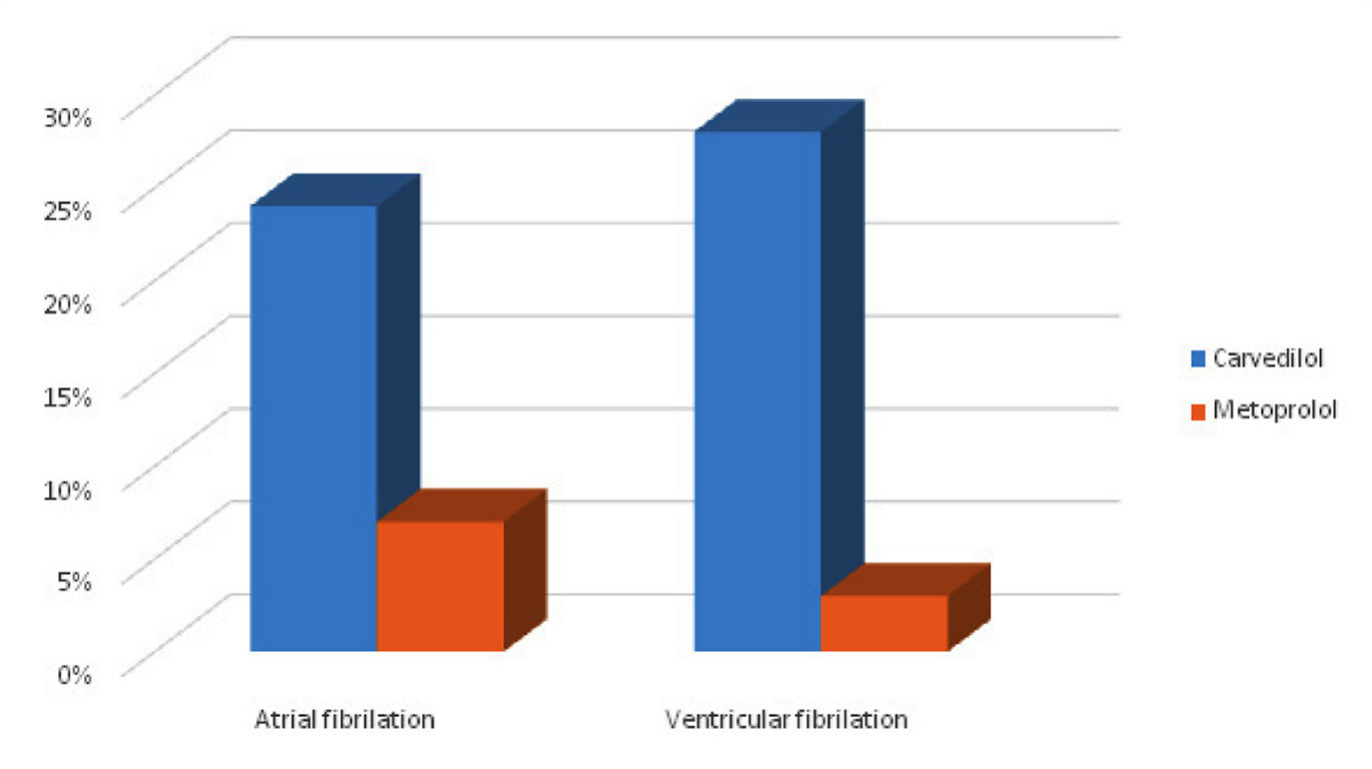
Post-Operative Arrhythmia in Carvedilol and Metoprolol Groups

Occurrence of atrial and ventricular fibrillation and maximum heart rate were recorded for both groups. Atrial fibrillation was seen in 18 patients in the carvedilol group and 21 ones in the metoprolol group (P = 0.577) (Figure 1). The results of Fisher exact test showed no significant relationship between the type of drug and occurrence of AF (Table 3). The highest prevalence of AF was recorded on the second and third days after the surgery; 24 patients had AF on the second post-operative day (9 in the carvedilol group and 15 in the metoprolol group) and 12 patients developed AF on the third day after the surgery (8 in the carvedilol group and 4 in the metoprolol group) (Table 4).

**Table 3.** Post-Operative Data in Carvedilol and Metoprolol Groups

Post-Operative	Carvedilol (n = 75)	Metoprolol (n = 75)	P value
**Atrial fibrillation**	18 (24%)	21 (28%)	0.577
**Ventricular fibrillation**	5 (7%)	2 (3%)	0.246
**Maximum heart rate**	204 ± 17	111 ± 36	0.126

**Table 4.** The Day of Atrial Fibrillation Incidence in Carvedilol and Metoprolol Groups

Day of AF Incidence	Carvedilol	Metoprolol
**First**	1	1
**Second**	9	15
**Third**	8	4
**> forth**	0	1

Among the study population, 69 patients had diabetes and AF was detected in 11 out of the 31 diabetic patients in the carvedilol group and in 9 out of the 38 ones in the metoprolol group (P = 0.283). Additionally, 132 patients were found to have HTN among whom, 17 out of the 67 patients in the carvedilol group and 19 out of the 65 patients in the metoprolol group experienced AF (P = 0.619). Comparison of the characteristics of the patients with and without AF showed that old age and maximum heart rate were significantly related to the occurrence of AF. Moreover, AF was recorded in 11 patients (14%) in the metoprolol group and 9 ones (12%) in the carvedilol group, with LVEF being between 35% and 45% (P=0.587) (Table 5).

**Table 5.** Comparing the Study Parameters between the Patients with and without Post-Operative Atrial Fibrillation

Study Parameters (Before, During, and After the Surgery)	With AF (n = 39)	Without AF (n = 111)	P value
**Age (year)**	62 ± 10	58 ± 10	0.044
**Male gender (n)**	19 (48%)	76 (68%)	0.028
**Hypertension (n)**	36 (92%)	96 (86%)	0.336
**Diabetes (n)**	20 (51%)	49 (44%)	0.442
**Previous MI (n)**	17 (44%)	39 (35%)	0.557
**Renal insufficiency (n)**	0 (0%)	4 (3.6%)	0.230
**Previous β blocker use (n**)	35 (89%)	106 (95%)	0.193
**Left ventricular ejection fraction**	44.7 ± 7	46.7 ± 7	0.147
**Left atrial diameter (mm)**	38.6 ± 4.2	38.7 ± 4.4	0.927
**Cross clamping time (min)**	47 ± 18	45 ± 21	0.698
**CPB time (min)**	79 ± 23	84 ± 45	0.587
**Ventricular fibrillation (n)**	5 (13%)	2 (2%)	0.005
**Maximum heart rate (n)**	121 ± 11	102 ± 31	< 0.001

## 5. Discussion

AF is the most common arrhythmia (20 - 40%) after CABG and usually occurs between the third and fifth days after the surgery (2). AF is associated with hemodynamic disturbances, systemic emboli, higher incidence of MI, and complications. In this regard, several preventive measures have been suggested and tried with various success rates, including β-blocker treatment before the surgery, amiodarone before the surgery, and pace placement after the surgery (11). Despite the improvements in techniques, myocardium preserve methods, and surgical experience, post-operative AF is still an important issue in heart surgery. Although the mechanism of post-surgical AF is not fully understood, mechanical damages to atrial structures, hypertension, increased volume, atrial ischemia due to the surgery, electrolyte imbalance, and pericardial injuries are some of the suggested involving factors (12, 13). In spite of the fact that the impact of these factors on AF incidence is controversial, it has been approved that inflammation and oxidative injury to atrial tissue is higher during the surgery (14-17). It has been shown that serum level of C-Reactive Protein (CRP) is significantly higher in the patients with post-operative AF. Moreover, prophylaxis effect of ascorbic acid and statins for post- operative AF has been already confirmed. All these findings indicate the importance of inflammation and oxidative injury in occurrence of AF after the surgery. In addition, several studies have shown age, β-blocker use, and heart failure as relative but independent factors for post-surgical AF (18-20). DM, pericardial effusion, pre-operative arrhythmias, repeated heart surgeries, and off- pump bypass surgery have also been suggested as linear, independent factors (21, 22). Β-blockers are effective medications for prophylaxis and have lower risk in comparison to other anti-arrhythmic medications (5, 6).

Carvedilol is a non-selective blocker of beta and alpha-1 receptors which decreases the levels of cardiac norepinephrine and increases parasympathic force of the heart. An additional characteristic of this drug compared to other beta blockers is its anti-inflammation and antioxidant characteristics making it potentially more effective in prevention of AF. Carvedilol is commonly used in the patients with congestive heart failure and post-MI (1, 16, 22, 23). On the other hand, this drug is an antagonist of sodium rapid depolarization canals and type 1 sodium canals thereby having similar effects as amiodarone (7, 16, 23). Anti-oxidant properties of carvedilol are applied through raising the levels of super-oxide dismutase and glutation synthetize enzymes. It also causes a decline in CRP level. However, metoprolol has not shown any of these effects.

Katritisis et al. compared the effects of carvedilol and bisoprolol on maintaining the sinus rhythm in 90 patients with drug-resistance AF whose rhythms were treated using cardioversion. The incidence of repeated AF was 14% lower in the patients treated with carvedilol although the difference was not statistically significant (P = 0.66). Nevertheless, Yoshoika et al. in a retrospective study compared

31 patients in the carvedilol group to 22 controls and found no significant difference between the two regarding the incidence of post-operative AF. Sadik Acikel et al. carried out a study on 110 patients and reported that carvedilol was superior to metoprolol in prevention of post-operative AF.

In the present study, the incidence of post-operative AF was reported as 24% in the carvedilol group and 28% in the metoprolol group, but the difference was not statistically significant. Besides, no significant relationship was found between diabetes and hypertension and the incidence of AF (P = 0.283 and P = 0.619, respectively). The only factor which was significantly related to the incidence of AF was old age. According to the study findings, carvedilol was not different from metoprolol in decreasing the incidence of post-CABG surgery AF. Yet, further studies are needed to confirm the preference of carvedilol to metoprolol and other beta blockers.

### 5.1. Limitations

The relatively small sample size and short follow-up period were some of the limitations of the present study. Moreover, the type of the beta blockers used before the surgery was not in control of the researchers and many of the patients had taken metoprolol until the day of surgery.

### 5.2. Suggestions

Given the anti-oxidant and anti-inflammatory effects of carvedilol, using it might be more beneficial in comparison to metoprolol, particularly in the patients with lower ejection fractions or heart failure. However, it seems that there is no significant difference between carvedilol and metoprolol in prevention of atrial fibrillation in the patients with normal ejection fractions. Yet, further researches are required to be conducted on the issue.
